# Analysis of the regional distribution of road traffic mortality and associated factors in Japan

**DOI:** 10.1186/s40621-021-00356-4

**Published:** 2021-10-28

**Authors:** Tasuku Okui, Jinsang Park

**Affiliations:** 1 Medical Information Center, Kyushu University Hospital, Maidashi 3-1-1, Higashi-ku, Fukuoka City, Fukuoka Prefecture 812-8582 Japan; 2 Department of Pharmaceutical Sciences, International University of Health and Welfare, Fukuoka, Japan

**Keywords:** Traffic crashes, Japan, Mortality, Census, Vital Statistics

## Abstract

**Background:**

Regional differences in road traffic (RT) mortality among municipalities have not been revealed in Japan. Further, the association between RT mortality and regional socioeconomic characteristics has not been investigated. We analyzed geographic differences in RT mortality and its associated factors using the Vital Statistics in Japan.

**Methods:**

We used data on RT mortality by sex and municipality in Japan from 2013 to 2017. We calculated the standardized mortality ratio (SMR) of RT for each municipality by sex using an Empirical Bayes method. The SMRs were mapped onto a map of Japan to show the geographic differences. In addition, an ecological study investigated the municipal characteristics associated with the SMR using demographic socioeconomic, medical, weather, and vehicular characteristics as explanatory variables. The ecological study used a spatial statistical model.

**Results:**

The mapping revealed that the number of municipalities with a high SMR of RT (SMR > 2) was larger in men than in women. In addition, SMRs of capital areas (Kanagawa and Tokyo prefectures) tended to be low in men and women. The regression analysis revealed that population density was negatively associated with the SMR in men and women, and the degree of the association was the largest among explanatory variables. In contrast, there was a positive association between the proportion of non-Japanese persons and SMR. The proportions of lower educational level (elementary school or junior high school graduates), agriculture, forestry, and fisheries workers, service workers, and blue-collar workers were positively associated with the SMR in men. The proportion of unemployed persons was negatively associated with the SMR in men.

**Conclusions:**

Socioeconomic characteristics are associated with geographic differences in RT mortality particularly in men. The results suggested preventive measures targeted at men of low socioeconomic status and non-Japanese persons are needed to decrease RT mortality further.

**Supplementary Information:**

The online version contains supplementary material available at 10.1186/s40621-021-00356-4.

## Background

Road traffic (RT) crashes are one of the leading causes of death globally, particularly in developing countries (Nantulya and Reich 2002; Staton et al. 2016). In Japan, RT mortality has steadily decreased over recent decades (Ministry of Health, Labour and Welfare of Japan 2021a), and it is approximately a few thousand annually. In Japan in 2020, total traffic mortality was 3718, and RT mortality was 3259 (87.7% of all traffic mortality) (Ministry of Health, Labour and Welfare of Japan 2021a). Legislation banning alcohol and driving has decreased the incidence and mortality of traffic accidents (Nishitani 2019; Nagata et al. 2008). In addition, safety innovations in cars and emergency medical care contributed to the decrease in traffic deaths (Oguchi 2016). Although some attributes of victims of RT crashes in Japan are unknown, studies have shown, for example, that male smokers and elderly drivers have an increased risk of traffic deaths (Igarashi et al. 2019; Matsuyama et al. 2018). However, few studies investigated the association between RT mortality and the socioeconomic characteristics of victims or crash sites in Japan. The socioeconomic characteristics of regions or drivers in other countries have been related to RT mortality (Jones et al. 2008; La Torre et al. 2007; Noland and Laham 2018; Rivas-Ruiz et al. 2007; Harper et al. 2015; Girasek and Taylor 2010). If RT mortality in Japan also has socioeconomic disparities, administrative measures in specific regions or targeting persons with low socioeconomic status might effectively prevent RT crashes.

The number of RT deaths is not large in Japan, and an epidemiological study investigating an association between socioeconomic status and RT mortality using individual data is difficult. In contrast, a common method for investigating an association between socioeconomic factors and RT mortality is to investigate regional differences in RT mortality. There are some studies investigating differences in RT mortality among prefectures in Japan (Inada et al. 2019; Nakamura 1984). The prefectures with high RT mortality are known in Japan (Shiomi 2019), and RT mortality rates tend to be low in capital regions (Nakamura 1984). However, RT mortality is largely affected by age, and an analysis taking into account of differences in age distribution among regions is needed. In addition, there have been no nationwide studies investigating differences in RT mortality between municipalities in Japan. There are high and low-risk regions for RT mortality in each prefecture, and it is useful to analyze the differences between municipalities. Moreover, there is no study investigating factors associated with RT mortality using various regional characteristics in Japan. In other countries, factors associated with RT mortality have been shown using region-specific data by ecological studies (Jones et al. 2008; La Torre et al. 2007; Rivas-Ruiz et al. 2007; Haghighi et al. 2020).

In this study, we investigated geographic differences in RT mortality and associated factors using data from the Vital Statistics in Japan.

## Methods

We extracted data on RT mortality by sex and municipality, and mortality data by sex and age group in Japan from 2013 to 2017 from the Vital Statistics in Japan (Ministry of Health, Labour and Welfare of Japan 2021a). The International Statistical Classification of Diseases and Related Health Problems 10th revision codes corresponding to RT mortality are V01–V98. Our mortality data include only traffic crashes that occurred on the road, and traffic crashes that occurred in places, such as parking spaces or on the water, are not included. Deaths of RT crash victims transported to hospitals are also included in the data. We obtained population data by age group, sex, and municipality from 2013 to 2017 from the data on “population, population demographics, and the number of households based on the Basic Resident Registry” (Ministry of Internal Affairs and Communications 2021a).

Table 1 shows the municipal characteristics used to investigate an association with RT mortality.

**Table 1:** Municipal characteristics used in this study

Characteristics	Description	Source
Demographic characteristics		
Population density	Number of persons per hectare	The Survey on Areas by Municipalities and the Basic Resident Register
Daytime population	Number of persons in daytime in a municipality	The Census
Proportion of young population	Proportion of young population aged below 15 years	The Basic Resident Register
Proportion of non-Japanese persons	Proportion of non-Japanese persons among the total population (%)	The Basic Resident Register
Proportion of divorced persons	Proportion of divorced persons among persons aged 15 years old or more (%)	The Census
Socioeconomic characteristics		
Proportion of persons with lower educational level	Proportion of elementary school or junior high school graduates among population aged 15 years or more (%)	The Census
Proportion of clerical workers	Proportion of clerical workers in the labor force (%)	The Census
Proportion of agriculture, forestry, and fisheries workers	Proportion of agriculture, forestry, and fisheries workers in the labor force (%)	The Census
Proportion of service workers	Proportion of service workers in the labor force (%)	The Census
Proportion of blue-collar workers	Proportion of blue-collar workers in the labor force (%)	The Census
Proportion of unemployed persons	Proportion of unemployed persons in the labor force (%)	The Census
Taxable income per capita	–	The Survey on Taxation Status of Municipal Tax and the Basic Resident Register
Medical characteristics		
Number of clinics per 100,000 persons	Number of clinics per 100,000 persons	The Survey of Medical Institutions and the Basic Resident Register
Number of physicians per 100,000 persons	Number of physicians per 100,000 persons	The Statistics of Physicians, Dentists and Pharmacists and the Basic Resident Register
Number of hospitals per 100,000 persons	Number of hospitals per 100,000 persons	The Survey of Medical Institutions and the Basic Resident Register
Weather characteristics		
Proportion of rainy days	Proportion of rainy days in a year (%)	Japan Meteorological Agency
Proportion of snow days	Proportion of snow days in a year (%)	Japan Meteorological Agency
Characteristics related to vehicles		
Proportion of holders of a driver’s license	Proportion of holders of a driver’s license among persons aged > 15 years (%)	The Statistics of Driver’s License and the Census
Number of owned vehicles per household	Number of owned vehicles per household among two-person households	The National Survey of Family Income and Expenditure
Number of owned motorcycles per 1000 persons	Number of owned motorcycles per household among 1000 persons	The Automobile Inspection & Registration Information Association and the Basic Resident Register

We obtained all data from the window of government statistics in Japan except for the proportion of divorced persons, the number of driver’s license holders, and the number of owned motorcycles (Ministry of Internal Affairs and Communications 2021b). We obtained data on the proportion of divorced persons from the Census (Ministry of Internal Affairs and Communications 2021c) and the number of driver’s license holders from the National Police Agency drivers’ license statistics (National Police Agency 2021). Data on the number of owned motorcycles were obtained from the Automobile Inspection & Registration Information Association (2021). In addition, the variables related to weather characteristics and characteristics related to vehicles were available for each prefecture but not by the municipality. Moreover, we used all data from 2015 in the analysis except for the number of physicians, the number of owned vehicles per household, and the proportion of persons with lower educational level. We used the number of physicians, the number of owned vehicles per household from 2014 in the analysis, because they were not available from 2015. Additionally, data on the educational level were not investigated in the Census in 2015, and thus the data from 2010 were used for the proportion of persons with lower educational levels. Map data of municipalities in Japan were obtained from the administrative district data of the digital national land information from the Ministry of Land, Infrastructure, Transport, and Tourism (2021).

We calculated the mortality rate by sex and age group using RT mortality and population data in Japan from 2013 to 2017. Then, by multiplying the mortality rate and population by age group for each municipality, we calculated the expected RT mortality for each municipality by sex. From the expected and observed mortality, we calculated the standardized mortality ratio (SMR) of RT crashes for each municipality by sex using an Empirical Bayes method (Clayton and Kaldor 1987). The SMRs were mapped onto a map of Japan to show the geographical differences in the SMR of RT crashes.

We investigated municipal characteristics associated with the SMR with an ecological study. In addition, the SMRs were log-transformed in the analysis and the outcome and explanatory variables were scaled. We used a spatial conditional autoregressive model for regression analysis (Ver Hoef et al. 2017) to consider the spatial correlation between adjacent municipalities. Only municipalities adjacent to other municipalities can be used in the spatial regression analysis. We conducted a multivariate analysis using all the explanatory variables in the regression analysis. The standardized partial regression coefficient (SPRC), 95% confidence interval (CI), and *p* value were calculated for each explanatory variable. A *p* value less than 0.05 was considered statistically significant. All statistical analyses were conducted using R3.6.3 (https://www.R-project.org/).

## Results

**Fig. 1:**
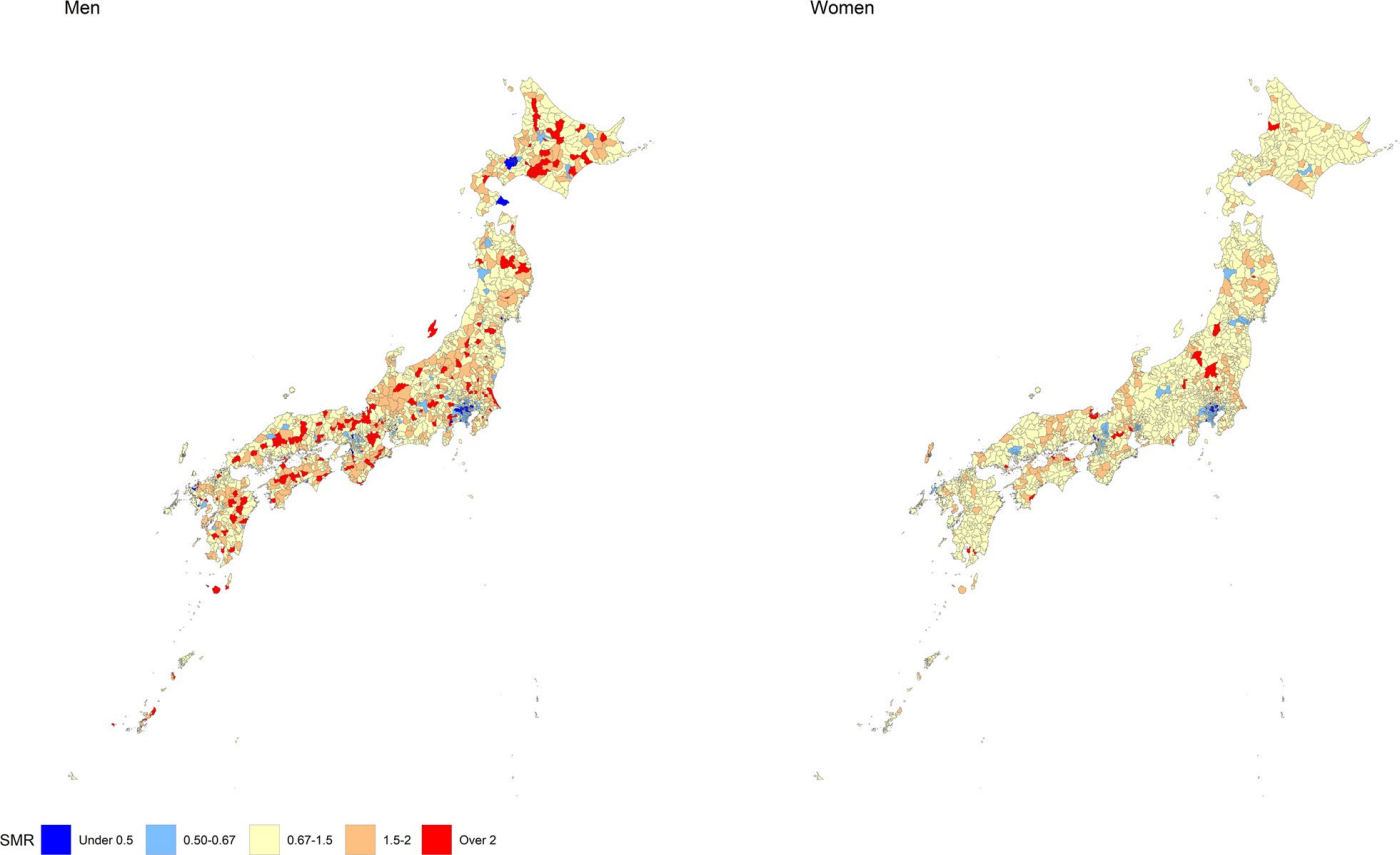
Geographic distribution of SMR of RT for men and women

Figure 1 shows the geographic distribution of SMR of RT crashes for men and women in all 1741 municipalities. Number of municipalities with a high SMR of RT (SMR > 2) was larger in men than in women. In addition, SMRs of capital areas (Kanagawa and Tokyo prefectures) tended to be low in men and women.

Table 2 shows municipalities with the highest SMR of RT crashes and their SMR for men and women. The municipalities had a particularly high SMR of RT crashes in men. Few overlaps were noted in the results of the 10 most populous municipalities for SMR between men and women.

**Table 2:** Municipalities with the highest SMR of RTA and their SMR for men and women

Rank	Men		Women	
	Municipality name (Prefecture name)	SMR	Municipality name (Prefecture name)	SMR
1	Onna village (Okinawa)	4.30	Uonuma city (Niigata)	2.38
2	Doshi village (Yamanashi)	4.17	Kuroshio town (Kochi)	2.29
3	Tobishima village (Aichi)	3.68	Oyama city (Tochigi)	2.27
4	Shimukappu village (Hokkaido)	3.49	Kuwana city (Mie)	2.24
5	Kameyama city (Mie)	3.40	Naganohara town (Gunma)	2.20
6	Karuizawa town (Nagano)	3.30	Karuizawa town (Nagano)	2.19
7	Ikusaka village (Nagano)	3.24	Maizuru city (Kyoto)	2.16
8	Ibaraki town (Ibaraki)	3.18	Yanai city (Yamaguchi)	2.14
9	Nanbu town (Yamanashi)	3.15	Tarumizu city (Kagoshima)	2.13
10	Atsuma town (Hokkaido)	3.13	Koka city (Shiga)	2.13

*SMR* standardized mortality ratio

Table 3 shows summary values of municipal characteristics. After excluding municipalities where the Census data are not fully available and municipalities that are not adjacent to other municipalities, 1687 municipalities were used in the regression analysis. The correlation coefficient matrix of the municipal characteristics is shown in Additional file 1.

**Table 3:** Summary values of municipal characteristics

Characteristics	Median (interquartile range)
	(*N* = 1687)
Demographic characteristics	
Population density	2.1 (0.6–8.2)
Daytime population	24,071.0 (8478.5–62,004.0)
Proportion of young population	12.0 (10.5–13.4)
Proportion of non-Japanese persons	0.7 (0.4–1.3)
Proportion of divorced persons	5.0 (4.3–5.8)
Socioeconomic characteristics	
Proportion of persons with lower educational level	22.7 (16.3–30.4)
Proportion of clerical workers	15.4 (13.3–17.6)
Proportion of agriculture, forestry, and fisheries workers	6.7 (2.3–14.4)
Proportion of service workers	1.1 (0.9–1.3)
Proportion of blue-collar workers	7.3 (6.5–8.4)
Proportion of unemployed persons	3.9 (3.3–4.7)
Taxable income per capita (1000 yen)	1103.3 (931.3–1293.0)
Medical characteristics	
Number of clinics per 100,000 persons	68.4 (54.1–84.2)
Number of physicians per 100,000 persons	128.8 (74.4–194.1)
Number of hospitals per 100,000 persons	6.0 (2.1–10.1)
Weather characteristics	
Proportion of rainy days	33.4 (31.0–38.6)
Proportion of snow days	5.2 (3.0–20.0)
Characteristics related to vehicles	
Proportion of holders of a driver’s license	75.7 (71.6–78.0)
Number of owned vehicles per household	1.6 (1.3–1.9)
Number of owned motorcycles per 1000 persons	26.0 (22.5–28.5)
Mortality rate	
Male RT mortality rate per 100,000 persons	6.7 (3.7–11.0)
Female RT mortality rate per 100,000 persons	2.8 (1.0–5.2)

*RT* road traffic

Table 4 shows the results of the regression analysis. Population density was negatively associated with the SMR in men and women, and daytime population was also negatively associated with the SMR in women. The proportion of non-Japanese persons was positively associated with the SMR in men and women. The absolute value of the SPRC for population density was the largest among the explanatory variables. The proportion of persons with lower educational level was positively associated with the SMR in men. The proportion of clerical workers was negatively associated with the SMR in women, and the proportion of workers in agriculture, forestry and fisheries, service, and blue-collar workers was positively associated with the SMR in men. In contrast, there was a negative association between the proportion of unemployed persons and taxable income per capita with men’s SMR. Also, the numbers of physicians and hospitals per 100,000 persons were positively associated with the SMR only in men. The proportion of rainy days was positively associated with the SMR only in women. The proportion of snow days was negatively associated with the SMR both in men and women. The number of owned vehicles per household was positively associated with the SMR in women.

**Table 4:** Results of the regression analysis

Explanatory variables	Men		Women	
	SPRC (95% CI)	*p* value	SPRC (95% CI)	*p* value
Demographic characteristics				
Population density	− 0.389 (− 0.455, − 0.323)	0.000	− 0.395 (− 0.460, − 0.329)	0.000
Daytime population	− 0.038 (− 0.083, 0.007)	0.100	− 0.102 (− 0.147, − 0.057)	0.000
Proportion of young population	− 0.006 (− 0.060, 0.049)	0.832	0.008 (− 0.046, 0.062)	0.774
Proportion of non-Japanese persons	0.090 (0.040, 0.140)	0.000	0.085 (0.036, 0.134)	0.001
Proportion of divorced persons	− 0.005 (− 0.061, 0.051)	0.858	0.005 (− 0.050, 0.061)	0.850
Socioeconomic characteristics				
Proportion of persons with lower educational level	0.084 (0.004, 0.163)	0.039	0.009 (− 0.070, 0.087)	0.831
Proportion of clerical workers	− 0.015 (− 0.089, 0.059)	0.691	− 0.092 (− 0.165, − 0.019)	0.014
Proportion of agriculture, forestry, and fisheries workers	0.105 (0.034, 0.177)	0.004	0.035 (− 0.036, 0.106)	0.332
Proportion of service workers	0.051 (0.008, 0.094)	0.020	0.019 (− 0.024, 0.061)	0.388
Proportion of blue-collar workers	0.069 (0.017, 0.121)	0.010	0.041 (− 0.010, 0.093)	0.118
Proportion of unemployed persons	− 0.066 (− 0.121, − 0.010)	0.020	− 0.023 (− 0.078, 0.032)	0.418
Taxable income per capita	− 0.027 (− 0.101, 0.047)	0.476	0.041 (− 0.032, 0.115)	0.270
Medical characteristics				
Number of clinics per 100,000 persons	0.040 (− 0.007, 0.087)	0.095	0.020 (− 0.027, 0.067)	0.402
Number of physicians per 100,000 persons	0.071 (0.024, 0.118)	0.003	− 0.006 (− 0.052, 0.041)	0.803
Number of hospitals per 100,000 persons	0.047 (0.003, 0.091)	0.036	0.028 (− 0.015, 0.072)	0.204
Weather characteristics				
Proportion of rainy days	− 0.026 (− 0.076, 0.025)	0.314	0.062 (0.013, 0.112)	0.014
Proportion of snow days	− 0.100 (− 0.170, − 0.031)	0.005	− 0.128 (− 0.197, − 0.059)	0.000
Characteristics related to vehicles				
Proportion of holders of a driver’s license	0.013 (− 0.112, 0.137)	0.844	0.013 (− 0.110, 0.137)	0.830
Number of owned vehicles per household	0.082 (− 0.044, 0.208)	0.201	0.156 (0.032, 0.280)	0.014
Number of owned motorcycles per 1000 persons	0.013 (− 0.031, 0.056)	0.576	− 0.023 (− 0.066, 0.020)	0.298

*SPRC* standardized partial regression coefficient, *CI* confidence interval

## Discussion

We found geographic differences in RT mortality in Japan and identified some characteristics associated with the SMR. There were few overlaps in the results of the 10 most populous municipalities for SMR between men and women, and one possible reason is that municipal characteristics related to SMR differ by sex. Actually, socioeconomic characteristics tended to be associated more with SMR in men than in women. We discuss the associations with identified predictors and the RT mortality below.

Population density was negatively associated with RT mortality for men and women, and daytime population was also negatively associated with the SMR in women. Urban areas and high population density are often associated with a decreased RT mortality (Nakamura 1984; Eksler et al. 2008; Huang et al. 2013; Liu et al. 2012). In urban areas, there are numerous medical institutions, and roads are well-maintained (Nakamura 1984). In addition, public transportation other than cars is generally available. Moreover, it is said that traffic crashes tend to be at a lower speed in urban areas; therefore, the injury risk is lower (Eksler et al. 2008; Borrell et al. 2005). Although it is considered that an increase in daytime population leads to an increase in traffic volume in a municipality, an increase in population had a negative effect on the SMR, probably because population size is related to the degree of urbanization in a municipality.

The proportion of non-Japanese persons was positively associated with RT mortality. It is known that types of traffic violations caused by foreign drivers tend to be different depending on regions where foreigners came from Yoh et al. (2017). However, whether foreign drivers tend to cause traffic crashes or not is uncertain in Japan, and no studies investigated an association between RT mortality and non-Japanese persons in Japan. An association between RT crashes and foreigners is different depending on countries (Leviäkangas 1998; Redelmeier et al. 2011). In Finland, crashes risk of foreign drivers is reportedly higher than domestic drivers (Leviäkangas 1998), and lack of knowledge of traffic rules, insufficient winter-time driving skills, and attitudes toward traffic safety are pointed out as possible reasons (Leviäkangas 1998). In Japan, non-Japanese people have fewer opportunities to learn about traffic safety than Japanese people (Alsamarrai 2013), and possibly have some difficulties in reading traffic rules documents in Japanese.

Some types of occupations were positively associated with RT mortality in men. According to the Occupational and Industrial Aspects of the Report of Vital Statistics in Japan, the age-standardized mortality rates of traffic accidents among men are high in transport and machine operation workers, construction and mining workers, and agriculture, forestry, and fisheries workers (Ministry of Health, Labour and Welfare of Japan 2021b). Traffic exposure is high in these types of workers, particularly transport and machine operation workers. A common feature of blue-collar, service, agriculture, forestry, and fisheries jobs is that the educational requirements for the workers are relatively low (Tanaka and Kobayashi 2021). Low educational levels and income are associated with higher risks of traffic accidents in other countries (Borrell et al. 2005; Ghiasvand et al. 2020; Spoerri et al. 2011). A likely reason for the association between traffic crashes and socioeconomic status is that persons with low socioeconomic status cannot afford safety equipment and are more likely to drive less safe cars (Girasek and Taylor 2010; Borrell et al. 2005). Moreover, high blood alcohol concentration rates and lack of seatbelt wearing are reportedly higher among traffic accident victims with low educational levels (Braver 2003). Pedestrians with low educational levels are also associated with a higher risk of traffic accidents (Spoerri et al. 2011). The association between RT mortality and low socioeconomic status was observed particularly in men in this study, and the same phenomenon has occurred in Europe (Borrell et al. 2005). Japanese men are more likely to work in sectors related to increased RT mortality than women. Exposure to traffic is also different between men and women (Borrell et al. 2005). On the other hand, the proportion of unemployed persons was negatively associated with RT mortality in men, whereas the unemployment rate is a major indicator of low socioeconomic status. An economic recession or an increase in the unemployment rate are associated with a decrease in traffic fatalities in other countries (Wegman et al. 2017; Lloyd et al. 2015), possibly because car speed and traffic volumes decrease when the unemployment rates rise.

Regarding other characteristics, there was a positive association between the numbers of physicians and hospitals per 100,000 persons and RT mortality. Although the reason is uncertain, it may be that the cause of death tends to be classified as a traffic crash with an autopsy in municipalities with high medical resources. The proportion of autopsies of decedents varies depending on regions in Japan (Matsubara 2020). The proportion of senility as a cause of death (one of the unknown causes) tends to be small in regions where the proportion of hospital deaths is large in Japan (Hasegawa 2017), and number of hospitals per capita is related to determination of cause of death in Japan.

Regarding weather characteristics, the traffic accident risk is higher on rainy days. In a study in Korea, female drivers and heavy rain are associated with the level of accident severity (Lee et al. 2018). On the other hand, traffic volumes reportedly decrease in areas with a high proportion of snow days. Moreover, the number of owned vehicles per household increases opportunities to drive a car, particularly women.

This study revealed municipalities with a high SMR of RT crashes in Japan. Those municipalities should take measures to reduce the SMR. There are regional differences in road characteristics in Japan (Shiomi 2019). Certain municipalities may need to review common accident sites and road maintenance. In addition, this study associated socioeconomic characteristics with RT mortality in men. An educational traffic safety campaign targeted at these people is needed to decrease RT mortality. It is also important to investigate regional differences in seatbelt use or characteristics of vehicles to understand the reasons for the association with socioeconomic characteristics as is done in the United States (Molnar et al. 2012). Furthermore, as non-Japanese persons appear to have a higher risk of causing RT mortality, traffic safety education targeted at non-Japanese persons is key to reducing RT mortality, particularly as the non-Japanese population shows an increasing trend in Japan (Ministry of Internal Affairs and Communications 2021a).

There are some limitations to this study. Firstly, we could not obtain RT mortality data disaggregated by drivers, pedestrian status, bicycle riders, and other data categories on the characteristics of vehicles or causes of accidents. Analyzing RT mortality by these factors might better understand the association between socioeconomic characteristics and RT mortality. Similarly, it is important to collect RT crashes incidence data by municipality in the future as this would enable the analysis of RT crashes incidence and survival. Secondly, this study is ecological, and an ecological fallacy might exist in the results. A study using individual data would be useful in verifying the results of this study. Thirdly, data on weather and vehicular characteristics cannot be obtained for each municipality, which may have affected the results. Fourth, socioeconomic characteristics investigated were related to socioeconomic characteristics of individuals. We did not investigate differences in road conditions or vehicle characteristics among municipalities because municipality-specific data on those characteristics are not publicly available. A study investigating regional differences in these characteristics is also warranted.

## Conclusions

We revealed geographic differences in RT mortality and its associated factors using the Vital Statistics in Japan. Our results show that municipalities with a high SMR tended to be observed more often in men. The SMRs of capital areas tended to be low both in men and women. Spatial regression analysis revealed that population density was negatively associated with the SMR in men and women, and the degree of the association was the largest among the explanatory variables. In contrast, there was a positive association between the proportion of non-Japanese persons and the SMR in men and women. In addition, the proportions of persons with lower educational level, agriculture, forestry, and fisheries workers, service workers, and blue-collar workers were positively associated with the SMR in men. On the other hand, the proportion of unemployed persons was negatively associated with men’s SMR. Therefore, we find that, in addition to demographic characteristics, socioeconomic characteristics of regions are associated with geographic differences in RT mortality particularly in men.

## Supplementary Information

**Additional file 1.** Correlation matrix for the explanatory variables.

## Data Availability

All the data used in this study can be obtained from publicly available statistics in Japan. The data sources are recorded in the Reference section.

## Abbreviations

RT: Road traffic
CI: Confidence intervals or credible intervals
SMR: Standardized mortality ratio
SPRC: Standardized partial regression coefficient
IQR: Interquartile range
