# Supplementary material for: Analysis of the regional distribution of road traffic mortality and associated factors in Japan
Source: Inj Epidemiol. 2021 Oct 28;8:60. doi: 10.1186/s40621-021-00356-4 (PMC8555252; doi:10.1186/s40621-021-00356-4)
Supplement: Supplementary file 1 — Additional file 1. Correlation matrix for the explanatory variables. [file 40621_2021_356_MOESM1_ESM.docx]

Supplementary table 1. The explanatory variables and variable number

| Variable number | Characteristics |
| --- | --- |
| 1 | Population density |
| 2 | Daytime population |
| 3 | Proportion of young population |
| 4 | Proportion of non-Japanese persons |
| 5 | Proportion of divorced persons |
| 6 | Proportion of persons with lower educational level |
| 7 | Proportion of clerical workers |
| 8 | Proportion of agriculture, forestry, and fisheries workers |
| 9 | Proportion of service workers |
| 10 | Proportion of blue-collar workers |
| 11 | Proportion of unemployed persons |
| 12 | Taxable income per capita |
| 13 | Number of clinics per 100,000 persons |
| 14 | Number of physicians per 100,000 persons |
| 15 | Number of hospitals per 100,000 persons |
| 16 | Proportion of rainy days |
| 17 | Proportion of snow days |
| 18 | Proportion of holders of a driver’s license |
| 19 | Number of owned vehicles per household |
| 20 | Number of owned motorcycles per 1,000 persons |

Supplementary table 2. Correlation coefficient matrix between the explanatory variables

| Variable number | 1 | 2 | 3 | 4 | 5 | 6 | 7 | 8 | 9 | 10 | 11 | 12 | 13 | 14 | 15 | 16 | 17 | 18 | 19 | 20 |
| --- | --- | --- | --- | --- | --- | --- | --- | --- | --- | --- | --- | --- | --- | --- | --- | --- | --- | --- | --- | --- |
| 1 | 1.00 | 0.73 | 0.60 | 0.48 | 0.01 | -0.82 | 0.67 | -0.81 | -0.18 | -0.37 | 0.38 | 0.67 | -0.05 | 0.39 | -0.03 | -0.34 | -0.42 | -0.02 | -0.26 | 0.26 |
| 2 |  | 1.00 | 0.50 | 0.43 | 0.11 | -0.69 | 0.55 | -0.66 | -0.11 | -0.34 | 0.28 | 0.60 | 0.07 | 0.60 | 0.19 | -0.23 | -0.27 | -0.03 | -0.17 | 0.16 |
| 3 |  |  | 1.00 | 0.31 | 0.03 | -0.59 | 0.43 | -0.46 | -0.21 | -0.31 | 0.03 | 0.52 | -0.12 | 0.21 | -0.04 | -0.11 | -0.22 | 0.14 | -0.02 | 0.15 |
| 4 |  |  |  | 1.00 | -0.21 | -0.45 | 0.36 | -0.47 | -0.09 | -0.22 | -0.08 | 0.59 | -0.05 | 0.16 | -0.12 | -0.30 | -0.20 | 0.19 | -0.03 | 0.13 |
| 5 |  |  |  |  | 1.00 | 0.10 | -0.18 | 0.04 | 0.19 | 0.16 | 0.46 | -0.31 | 0.07 | 0.24 | 0.36 | 0.12 | -0.18 | -0.18 | -0.14 | 0.10 |
| 6 |  |  |  |  |  | 1.00 | -0.70 | 0.73 | 0.24 | 0.47 | -0.21 | -0.75 | -0.02 | -0.39 | 0.02 | 0.30 | 0.29 | 0.05 | 0.26 | -0.18 |
| 7 |  |  |  |  |  |  | 1.00 | -0.78 | -0.16 | -0.28 | 0.17 | 0.69 | 0.13 | 0.37 | -0.06 | -0.22 | -0.23 | -0.04 | -0.23 | 0.17 |
| 8 |  |  |  |  |  |  |  | 1.00 | 0.06 | 0.20 | -0.32 | -0.69 | -0.05 | -0.40 | 0.03 | 0.29 | 0.28 | 0.04 | 0.25 | -0.23 |
| 9 |  |  |  |  |  |  |  |  | 1.00 | 0.16 | 0.07 | -0.25 | 0.04 | 0.02 | 0.11 | -0.03 | -0.07 | 0.11 | 0.10 | 0.07 |
| 10 |  |  |  |  |  |  |  |  |  | 1.00 | 0.04 | -0.30 | -0.16 | -0.26 | 0.01 | 0.08 | 0.20 | -0.06 | 0.02 | -0.04 |
| 11 |  |  |  |  |  |  |  |  |  |  | 1.00 | -0.07 | -0.04 | 0.18 | 0.12 | -0.20 | -0.30 | -0.14 | -0.20 | 0.17 |
| 12 |  |  |  |  |  |  |  |  |  |  |  | 1.00 | -0.07 | 0.26 | -0.09 | -0.23 | -0.08 | -0.02 | -0.22 | 0.19 |
| 13 |  |  |  |  |  |  |  |  |  |  |  |  | 1.00 | 0.37 | 0.01 | 0.04 | -0.09 | -0.02 | -0.01 | -0.01 |
| 14 |  |  |  |  |  |  |  |  |  |  |  |  |  | 1.00 | 0.52 | -0.07 | -0.22 | -0.02 | -0.09 | 0.10 |
| 15 |  |  |  |  |  |  |  |  |  |  |  |  |  |  | 1.00 | 0.10 | -0.09 | -0.08 | -0.04 | 0.03 |
| 16 |  |  |  |  |  |  |  |  |  |  |  |  |  |  |  | 1.00 | 0.45 | -0.26 | -0.01 | -0.40 |
| 17 |  |  |  |  |  |  |  |  |  |  |  |  |  |  |  |  | 1.00 | -0.19 | 0.14 | -0.49 |
| 18 |  |  |  |  |  |  |  |  |  |  |  |  |  |  |  |  |  | 1.00 | 0.83 | 0.11 |
| 19 |  |  |  |  |  |  |  |  |  |  |  |  |  |  |  |  |  |  | 1.00 | -0.12 |
| 20 |  |  |  |  |  |  |  |  |  |  |  |  |  |  |  |  |  |  |  | 1.00 |
